# Exploring Coaching Relationships, Teacher Self-Efficacy, and Motivation: Psychological Associations with Teachers’ Acceptability of Coaching and Stress

**DOI:** 10.3390/bs16060838

**Published:** 2026-05-22

**Authors:** Jiayi Wang, Duli Shi

**Affiliations:** 1 Department of Psychological, Health, and Learning Sciences, University of Houston, Houston, TX 77204, USA; 2 Communication Studies Department, New Mexico State University, Las Cruces, NM 88003, USA; shiduli@nmsu.edu

**Keywords:** coaching relationship, teacher self-efficacy, teachers’ motivation for coaching, acceptability of coaching, teacher stress

## Abstract

Teacher coaching is a widely used approach to support teacher professional development, yet the relational and psychological factors that influence coaching outcomes remain underexplored. Guided by the Expectancy-Value Theory and prior literature, this study examined the role of coaching relationships, teacher self-efficacy, and teachers’ motivation for coaching in the coaching process, with two key outcomes: teachers’ acceptability of coaching and perceived stress. A sample of 308 K-12 teachers from the United States completed relevant measures. Structural equation modeling revealed that high-quality coaching relationships were significantly associated with greater acceptability, self-efficacy, and motivation, particularly increased perceived benefits and reduced reservations. Coaching relationships and coaching acceptability were indirectly associated via self-efficacy and perceived benefits. The indirect association between coaching relationships and stress was fully explained through self-efficacy. These findings underscore the importance of fostering strong relationships with teachers and addressing motivational components to enhance the effectiveness of coaching interventions.

## 1. Introduction

Teacher coaching has been gaining increasing attention internationally as an effective approach to teacher education, supporting teachers in implementing interventions and better meeting students’ needs (Kochmanski, 2023; Reddy et al., 2022). Coaching is generally considered a type of teacher professional development (PD). However, unlike traditional PD, which tends to be broad and less personalized, teacher coaching offers a more intensive, individualized form of PD. It typically involves one-on-one collaboration between a coach and a teacher over an extended period, to enhance specific instructional practices within the context of the teacher’s classroom (Kraft et al., 2018).

A critical aspect of effective coaching is the quality of the relationships between coaches and teachers. In the consultation literature, relationship-building is viewed as a foundational component of successful professional learning (Frank & Kratochwill, 2014). Research has shown that collaborative, non-coercive relationships between consultants and teachers are associated with improved instructional practices (Davis et al., 2021; Newman et al., 2015). Similarly, studies in the coaching literature have demonstrated that supportive, collaborative relationships with coaches can enhance teachers’ motivation to apply instructional strategies learned from coaching to their teaching practices (Johnson et al., 2016; Pas et al., 2022).

Despite growing recognition of the importance of coaching relationships, less is known about the specific teacher outcomes such as teachers’ acceptability of coaching and teacher stress, which represent complementary dimensions of teachers’ experiences with coaching. Acceptability refers to the extent to which teachers perceive coaching strategies as appropriate, fair, and practical, and is considered a critical component of social validity in intervention research (Briesch et al., 2013; Kazdin, 1980). Research has found significant positive associations between teacher acceptability and intervention fidelity, underscoring the importance of teacher buy-in for the effectiveness of coaching initiatives (Dart et al., 2012). Meanwhile, given the high-stress nature of teaching, various studies have identified predictors and intervention strategies aimed at alleviating teacher stress (Herman et al., 2021; Jentsch et al., 2023). As acceptability reflects cognitive evaluation of coaching practices (Yin et al., 2025), whereas stress indicates the emotional strain often experienced in teaching. Examining both outcomes provides a more comprehensive picture of how coaching relationships are associated with not only teachers’ willingness to engage with and apply coaching strategies, but also their overall well-being.

Furthermore, limited research has examined the psychological factors associated with the relationship between coaching relationships and these outcomes. Unpacking these associations is especially important for teacher coaching practice as it helps identify the key pathways through which coaching can be improved and made more effective. In this study, we look into how two psychological constructs, teacher self-efficacy and motivation in coaching, provide potential pathways for the association between coaching relationship and teachers’ acceptability of coaching and teacher stress. Self-efficacy refers to individuals’ beliefs in their capacity to produce desired outcomes through their actions (Bandura, 1999, 2006). In education, teacher self-efficacy reflects confidence in managing classrooms, delivering instruction, and engaging students effectively (Calkins et al., 2024), and has been shown to buffer against stress while promoting professional engagement (Steigleder et al., 2023).

Teachers’ motivation for coaching is conceptualized through the lens of Expectancy-Value Theory (EVT; Wigfield & Eccles, 2000; Eccles & Wigfield, 2023). Within this framework, motivation is shaped by both expectancy for success and value beliefs. The expectancy component focuses on their anticipated success in future tasks (Urhahne & Wijnia, 2023). The value component of the framework consists of three types of value beliefs and three categories of costs that influence an individual’s decision to engage in or avoid a task (Eccles & Wigfield, 2023). The value beliefs include attainment value, intrinsic value, and utility value, reflecting how significant, enjoyable, and beneficial a task is perceived to be. On the other hand, costs encompass opportunity cost (the inability to engage in other activities due to task participation), effort cost (the level of exertion required to complete the task), and emotional cost (the potential psychological impact of engaging in the task). 

While research has documented the role of teacher self-efficacy and motivation in broader educational contexts (Skaalvik & Skaalvik, 2017; Sun & Yin, 2025), few studies have examined how these constructs function within the context of school coaching. Even fewer have investigated how they explain the indirect associations between coaching relationships and outcomes such as acceptability and stress. Addressing this gap, the present study aims to advance the field by exploring how these psychological constructs are associated with key teacher outcomes in the coaching process. Such knowledge will potentially contribute to more effective school coaching programs. 

**Figure 1:**
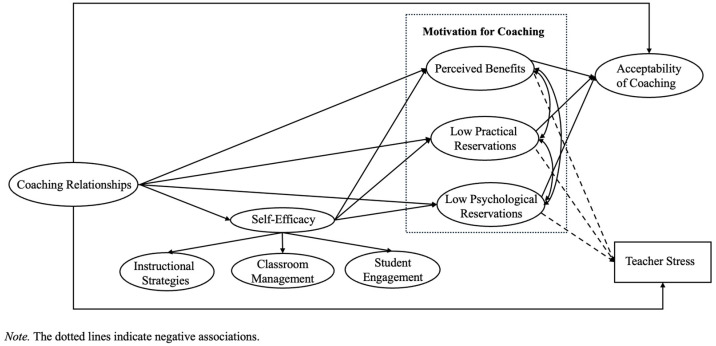
Conceptual Model.

Guided by EVT (Eccles & Wigfield, 2023) and prior literature, the present study examined the direct and indirect associations between coaching relationships and teachers’ acceptability of coaching and teacher stress among teachers in the United States. Particularly, we hypothesized that coaching relationships would be directly linked to both outcomes and that self-efficacy and motivation would be statistically linked to these relationships. To provide a more nuanced understanding of teacher motivation, we examined its three distinct dimensions: perceived benefits (aligned with expectancy for success and value beliefs), practical reservations (capturing opportunity and effort costs), and psychological reservations (reflecting emotional costs). Investigating these distinct dimensions allows us to account for the multifaceted nature of motivation and assess its complex effects. The conceptualized model is presented in Figure 1.

### 1.1. A Conceptual Framework for Teachers’ Motivation for Coaching

In this study, we build upon existing research examining the factors and outcomes associated with teacher motivation for coaching by developing a conceptual framework (see Figure 1) grounded in the EVT (Eccles & Wigfield, 2023; Wigfield & Eccles, 2000). The EVT provides a well-established lens for understanding the factors influencing individuals’ motivation and achievement-related choices and actions. It posits that socializers’ beliefs and behaviors influence an individual’s self-schema (e.g., self-efficacy), which then impacts motivation and choices of behaviors. In particular, socializer refers to significant others for the individual, such as peers, teachers, or supervisors. For example, when teachers have supportive relationships with and positive expectations of the student, the student’s self-efficacy is likely to be lifted, leading to higher motivation for tasks.

Applying this theoretical framework in the context of coaching, we explored how the teacher-coach relationship, an important socializing influence, is associated with teachers’ sense of self-efficacy and, in turn, their motivation to engage in coaching. Ultimately, these psychological mechanisms are likely related to teachers’ acceptability of coaching and their overall stress levels. As supported by prior research (e.g., Bandura, 1999; Zhang et al., 2021), coaching relationships can foster a trusting and constructive environment where teachers feel empowered to improve their instructional practices (Johnson et al., 2016).

Within the EVT, self-efficacy and motivation represent two key psychological mechanisms that may help explain how relational experiences with coaches are associated with teacher outcomes. Self-efficacy is a well-documented predictor that influences teachers’ tendency to engage in and benefit from coaching (Aytaç, 2021; Merle et al., 2023). Motivation is equally important, as it reflects the value teachers place on coaching and their willingness to invest in the process. We further examine three dimensions of coaching motivation: (a) perceived benefits, reflecting teachers’ expectation for success and value beliefs about the utility and significance of coaching; (b) practical reservations, referring to concerns about time and effort (i.e., opportunity and effort costs); and (c) psychological reservations, which relate to emotional costs (Wang et al., 2025a).

Overall, this study answers the prior calls to understand coaching through psychological underpinnings and interpersonal influence (Erchul, 2023) and contributes to the broader literature on teacher coaching by identifying specific relational, self-efficacy, and motivational factors that shape teachers’ acceptability in coaching and influence stress. By unpacking the relationships among these key components in coaching, we aim to provide insights into how coaching can be optimized to support teacher engagement and well-being. 

### 1.2. Associations of Coaching Relationships with Acceptability and Stress

The coaching relationship has been considered a critical component in coaching that affects teachers’ acceptability of coaching practices and learned skills (e.g., Pas et al., 2022; Wehby et al., 2012). Acceptability refers to teachers’ perception of an intervention as appropriate, fair, and practical, as well as their willingness to continue engaging in it with enthusiasm (Briesch et al., 2013; Kazdin, 1980). Given that strong teacher-coach relationships indicate mutual agreement on coaching goals and satisfaction with coaching (Ruble et al., 2023), high-quality coaching relationships not only foster motivation for coaching but also enhance teachers’ acceptability of using data to refine teaching approaches and interventions, ultimately benefiting student learning (Reddy et al., 2019, 2021). Despite these promising associations, there remains a notable gap in the literature, as few empirical studies have directly examined the association between coaching relationships and teachers’ acceptability of coaching.

In terms of teacher stress, support from social relationships, whether with students, colleagues, or professional support staff, plays a substantial role based on social support theory (Cohen & Wills, 1985). For example, positive relationships with coworkers have been linked to lower stress levels among teachers (Wang et al., 2025b). In Haydon et al.’s (2018) study, teachers indicated that supportive administrative relationships can be a protective factor against work-related stress. However, limited research has specifically explored how the teacher-coach relationship may help teachers cope with stress. Given the emotionally demanding nature of teaching and the potential for coaches to provide support, further investigation into this relationship is warranted.

### 1.3. Associations of Motivation with Acceptability and Stress

Within the context of school coaching, teachers’ motivation for coaching plays a critical role in shaping both engagement with the coaching process and the coaching outcomes. Teachers often exhibit varying levels of motivation to participate in coaching, which can significantly influence the effectiveness of coaching (Shernoff et al., 2017). Given its impact, motivational interviewing has been increasingly integrated into coaching models to foster teacher buy-in and facilitate meaningful behavioral change (Pas et al., 2022). A growing body of research supports the connection between teacher motivation for coaching or PD and key outcomes, including teacher acceptance (e.g., Osman & Warner, 2020; Owens et al., 2021). Teachers who are more motivated to engage in coaching are more likely to view coaching practices as useful and appropriate, and are thus more inclined to adopt and apply new instructional strategies in their classrooms (Owens et al., 2021). For instance, Palermo and Thomson (2019) found that among 119 U.S. teachers, motivation for professional development was a significant predictor of positive changes in instructional practices. Thus, we propose a positive association between teacher motivation with their acceptability of coaching. 

Comparatively, fewer studies have examined the association between teacher motivation for coaching and their well-being. However, in a recent study of 688 Italian teachers conducted during the COVID-19 pandemic, which involved an abrupt transition into online teaching, Toto and Limone (2021) found that higher levels of teacher motivation were significantly associated with lower levels of perceived stress. This finding suggests that motivation may serve not only as a driver of professional growth but also as a protective factor against occupational stress. It would be informative to examine how different dimensions of motivation, such as perceived benefits or emotional reservations, may be differentially related to stress levels.

### 1.4. Mediating Roles of Self-Efficacy and Motivation

While a few recent studies have highlighted the importance of coaching relationships in teachers’ acceptability and stress (e.g., Pas et al., 2022; Reddy et al., 2021), research on the underlying psychological factors remains limited. Emerging evidence from related areas of education suggests that both self-efficacy and motivation may function as mediators between relational support and outcome variables. For example, teacher self-efficacy has been shown to mediate the relationship between perceived social support and subjective well-being (Fu et al., 2022), as well as between teacher collaboration and emotional strain (Kingsford-Smith et al., 2023).

Motivation has also been identified as a key mediator in the relationship between social relationships or self-efficacy and various outcomes in the educational context. For instance, student motivation has been shown to mediate the relationship between self-efficacy and academic achievement (Teng et al., 2021), as well as between self-efficacy and satisfaction with learning (Doménech-Betoret et al., 2017). Although motivation has been studied as a mediating variable, these studies have largely focused on students, with limited attention to teacher motivation, especially in the context of coaching. 

According to the EVT, relational factors (e.g., coach-teacher alliance) first enhance an individual’s self-efficacy, which subsequently promotes motivation, ultimately shaping both behavioral and psychological outcomes (Eccles & Wigfield, 2023). Although this theoretical sequence is articulated, relatively few empirical studies have explicitly tested this pathway within teacher coaching contexts. Existing research, however, does consistently support a positive relationship between self-efficacy and motivation (Calkins et al., 2024; Zhang et al., 2021). Thus, the present study aims to empirically examine the sequential mediating roles of self-efficacy and motivation, providing nuanced insights into how coaching relationships contribute to positive teacher outcomes and experiences.

### 1.5. Direct Associations Among Relationship, Self-Efficacy, and Motivation

In the context of school coaching, there are direct associations among relational experiences, self-efficacy, and motivation. Research supports the role of positive working relationships in enhancing teacher self-efficacy. For example, Çoban et al. (2020) found that principals’ instructional leadership practices positively influenced teachers’ self-efficacy, both directly and indirectly, through fostering teacher collaboration. In this study, we aimed to capture a comprehensive view of teachers’ self-efficacy across three dimensions: delivering quality instructional strategies, effectively managing classroom behaviors, and engaging students in learning (Tschannen-Moran & Hoy, 2001).

The quality of teachers’ relationships with coaches also shapes teachers’ motivation for coaching. This extends the EVT by connecting relationship directly with motivation. Self-determination theory emphasizes that relatedness, the sense of connection and trust with others, is essential for fostering intrinsic motivation and psychological well-being (Ryan & Deci, 2000). Therefore, coaching relationships can be important in fostering motivation for professional learning. Specifically, Zhang et al. (2021) found that collegial support and administrative leadership, as a school climate factor, increased teacher motivation for professional development. Building on prior literature, we propose that positive teacher-coach relationships are directly associated with both greater self-efficacy and motivation for coaching, extending expectancy–value theory by examining these parallel associations beyond the established sequential pathway from self-efficacy to motivation.

## 2. Method

### 2.1. Procedures and Participants

The data used in this study were drawn from a larger dataset designed to examine various aspects of K-12 teachers’ attitudes and experiences with coaching in the United States. Data in the broader dataset was collected through Pollfish (2025), an online survey platform with access to a large participant pool. Eligible participants need to (a) be at least 18 years old and (b) currently work as a K-12 teacher in the United States. To ensure data quality, Pollfish employs multiple approaches, including respondent verification to confirm human engagement and the exclusion of random response patterns. Additional attention check items were embedded within the survey, and any responses failing these checks were excluded from the final dataset. All procedures for data collection and analysis were approved by the institutional review board.

The present study represents a more focused, cross-sectional analysis derived from that larger dataset. We used a subset of participants who self-reported having experience with coaching in order to examine coaching-specific variables. Participants were provided with a definition of coaching and asked to indicate whether they had such experience. Those without coaching experience (*n* = 217) were excluded, resulting in a final sample of 308 participants. Among these participants, 247 (80.2%) reported engaging in instructional coaching, 210 (68.2%) in behavioral coaching, and 157 (50.9%) in manualized intervention coaching. Regarding gender, 134 (43.5%) identified as female and 174 (56.5%) as male. Participants’ ages ranged from 20 to 69 years, with an average age of 37 (*SD* = 8.08). Teaching experience varied from 1 to 40 years, with a mean of 11 years (*SD* = 6.64). Regarding racial background, the majority of participants identified as White (*n* = 277, 89.9%), followed by Black or African American (*n* = 21, 6.8%), Hispanic or Latino (*n* = 4, 1.3%), and Asian (*n* = 3, 1.0%). The majority of participants reported working in public schools (*n* = 217, 70.5%), with 88 teachers (28.6%) working in private schools and 3 (1.0%) in other educational settings. In terms of grade level taught, 188 participants (58.8%) worked in secondary schools, 106 (34.4%) in elementary schools, and 18 (5.8%) in pre-kindergarten or kindergarten classrooms. Geographically, participants were located across 49 states in the United States, with the largest representation from California (*n* = 41), followed by New York (*n* = 24), Texas (*n* = 20), and Florida (*n* = 14). 

### 2.2. Measures

Participants filled out a demographic survey, which collected details about their gender identity, age, years of teaching, racial identity, and the state where they work. In addition to the demographic survey, participants completed the following validated measures.

### 2.3. Coach-Teacher Relationships

The Working Relationship subscale from the Coach-Teacher Alliance measure for teachers (Johnson et al., 2016) was used to understand the relationship quality between teacher and coach. This subscale includes six items (e.g., “The coach and I worked together collaboratively”), rated on a 5-point Likert scale ranging from “Never” to “Always.” In the current study, internal consistency reliability for this subscale was high (*α* = 0.87; *ω* = 0.88).

#### 2.3.1. Teacher Self-Efficacy 

Teacher Sense of Self-Efficacy Scale-Short Form (TSES; Tschannen-Moran & Hoy, 2001) was used to examine teachers’ self-efficacy in three areas, including instructional strategies, classroom management, and student engagement. The scale included twelve items rated on a 9-point scale ranging from “Nothing” to “A Great Deal.” Example items included: “To what extent can you craft good questions for your students?” and “How much can you do to calm a student who is disruptive and noisy?” In the current sample, reliability was acceptable to good across the subscales: instructional strategies (*α* = 0.70, *ω* = 0.71), classroom management (*α* = 0.75, *ω* = 0.76), and student engagement (*α* = 0.81, *ω* = 0.81). 

#### 2.3.2. Teacher’s Motivation Assessment for Coaching

Teacher’s Motivation Assessment for Coaching (T-MAC; Wang et al., 2025a) is a 20-item instrument designed building upon EVT to assess teachers’ motivation for participating in coaching. Participants were provided with a definition of teacher coaching to ensure a consistent understanding of the concept. They then rated each item on a 5-point scale from “Strongly Disagree” to “Strongly Agree.” Three subscales were assessed. Perceived benefits (e.g., “Coach helps me to identify my students’ needs”) is consistent with expectancy for success and task value components of EVT. Meanwhile, practical reservations (e.g., “The effort that it takes to participate in coaching is not worth the benefits”) and psychological reservations (e.g., “I am worried about being evaluated negatively by a coach”) capture perceived costs about engaging in coaching. Items on the practical and psychological reservations subscales were reverse-coded, meaning that higher scores indicated lower levels of reservation. We renamed these reserved factors to “low practice reservation” and “low psychological reservation”. Wang et al. (2025a) reported acceptable-to-strong reliability for each subscale: Perceived Benefits (*α* = 0.92, *ω* = 0.92), Psychological Reservations (*α* = 0.88, *ω* = 0.89), and Practical Reservations (*α* = 0.72, *ω* = 0.73). In the current study, similar levels of internal consistency were achieved: perceived benefits (*α* = 0.92, *ω* = 0.92), psychological reservations (*α* = 0.83, *ω* = 0.84), and practical reservations (*α* = 0.88, *ω* = 0.89).

#### 2.3.3. Teachers’ Acceptability of Coaching

The acceptability subscale from the Usage Rating Profile–Intervention Revised (URP-IR; Briesch et al., 2013) was used to assess teachers’ perceptions of the appropriateness, helpfulness, and appeal of coaching as an intervention. Participants were instructed to consider coaching as a form of intervention when rating items on a 6-point Likert scale ranging from 1 (Strongly Disagree) to 6 (Strongly Agree). There are nine items in this subscale and a sample item was “This intervention is a good way to handle the child’s behavior problem.” The acceptability subscale has demonstrated strong internal consistency in prior research (*α* = 0.95; Briesch et al., 2013) and the current teacher sample (*α* = 0.92; *ω* = 0.93).

#### 2.3.4. Teacher Stress

To assess teachers’ perceived stress levels regarding their job, we used a single-item teacher stress measure developed by Eddy et al. (2019). The item asked, “How stressful is your job?” and responses were rated on an 11-point Likert-like scale ranging from 0 (not stressful) to 10 (very stressful). This item has demonstrated convergent validity, showing a positive association with the emotional exhaustion subscale of Maslach Burnout Inventory-Educators Survey (Maslach et al., 1996), with Kendall’s tau (*τ*) values ranging from 0.31 to 0.45 in three time points (Eddy et al., 2019). Additionally, test–retest reliability was supported by a significant correlation between stress ratings at baseline and end of the study year, with *τ* = 0.58, indicating acceptable temporal stability (Eddy et al., 2019). The single-item measure of stress was selected to provide efficient and easy-to-understand feedback and reduce the respondent burden, given the large number of variables examined and the primary focus on evaluating the effects of coaching relationships.

### 2.4. Data Analysis

To test our hypothesized model of the coaching relationships and teachers’ acceptability of coaching and stress, we employed a two-step modeling approach using Mplus Diagrammer 1.8.11, which included a confirmatory factor analysis (CFA) model and a series of nested structural models. Mardia’s skewness and kurtosis tests were significant (*p* < 0.001), indicating multivariate non-normality. Thus, maximum likelihood estimation with Satorra-Bentler corrections (MLM estimator) was used to ensure robust standard error estimation and model evaluation. We conducted a post hoc power analysis using the semPower package in R (Moshagen & Bader, 2024) to assess whether the obtained sample size (*N* = 308) was adequate for the hypothesized structural equation model. The degrees of freedom (*df* = 1094) were derived from the Mplus SEM results, with an alpha level of 0.05, and H1 RMSEA = 0.05. The results showed that the achieved power exceeded 99.99% to detect global model misspecification, which also reflected the model complexity. Following commonly used cutoff values (Hu & Bentler, 1999; Marsh et al., 2005; Neff et al., 2019), model fit was assessed using the root mean square error of approximation (RMSEA; < 0.08), the comparative fit index (CFI; ≥0.95 for good, ≥0.90 for acceptable), the Tucker–Lewis index (TLI; ≥0.95 for good, ≥0.90 for acceptable), and the standardized root mean square residual (SRMR; <0.08). The chi-square statistics was considered more cautiously given its sensitivity to sample size and multivariate non-normality (Alavi et al., 2020). There was no missing data in the dataset received from Pollfish. We also included gender, age, race, and years of teaching as covariates to control for their associations with outcome variables.

In the measurement stage, CFA was conducted to validate the latent multi-indicator constructs, including coaching relationships, self-efficacy, three dimensions of motivation (i.e., perceived benefits, practical reservations, and psychological reservations), and acceptability to coaching. Stress was measured using a single indicator, so it is more vulnerable to measurement errors. Following Kenny’s (2012) recommendation, the factor loading was fixed to 1 and item residual variance was fixed to 2.848 (the observed indicator variance [6.781] multiplied by the assumed unreliability [1–0.58]), where 0.58 was based on Eddy et al.’s (2019) test–retest reliability for the single-item measure of stress. Self-efficacy was modeled as a second-order latent variable comprising three dimensions: instructional strategies, classroom management, and student engagement. All other variables (coaching relationship, perceived benefits, psychological reservation, practical reservation, and acceptability of coaching) were modeled as first-order variables with multiple observed indicators. Given the newly developed measure of teachers’ motivation in the coaching context (Wang et al., 2025a), our study examined how its three dimensions exert distinct effects; therefore, the constructs were modeled as first-order factors. All factor loadings for latent constructs were examined and ensured to be above 0.40. The measurement model builds a baseline model for testing our theoretically hypothesized model, which was more constrained. Following Kline’s (2016) model trimming and building approach, hypothesis testing was conducted using multiple chi-square difference tests to evaluate whether eliminating or adding free parameters resulted in a significant decrement in overall model fit. The three dimensions of motivation (i.e., perceived benefits, practical reservations, and psychological reservations) were allowed to covary in the model. The mediating roles of self-efficacy and motivation were tested through a bootstrapping procedure (*N* = 5000).

## 3. Results

### 3.1. Descriptive Statistics and Measurement Model

Descriptive statistics, including the mean, standard deviation, Cronbach’s *α*, composite reliability (CR), and average variance extracted (AVE) for all variables are reported in Table 1. All latent constructs demonstrated satisfactory reliability, as indicated by high Cronbach’s α and CR values exceeding 0.70. Majority AVE values were above 0.50, except for instructional strategies, which was slightly lower at 0.45. The separate CFA model validating the measurement of all factors exhibited acceptable fit: *χ*^2^ (962) = 1508.45, *p* < 0.001, CFI = 0.93, TLI = 0.92, RMSEA = 0.043, 90% CI [0.039, 0.047], SRMR = 0.059. Thus, the items measuring the hypothesize constructs conformed to the proposed factor structure. Guided by modification indices, the error terms of three pairs of items were sequentially allowed to correlate due to their similar wording. For example, the items “I feel nervous about being observed by the coach” and “I am worried the coach will judge me” used closely related emotional responses, justifying the correlation of their error terms. First, the items “I feel nervous about being observed by the coach” and “I am worried the coach will judge me” used closely related emotional responses and reflected semantic similarity between the words “nervous” and “worries,” justifying the correlation of their error terms. The items “I appreciate the coach’s help in addressing my concerns about students” and “The coach shares helpful materials and resources for teaching with me” are both general description of coaches’ help. The items “The coaching is a fair way to handle the child’s behavior problem” and “Coaching is a good way to handle the child’s behavior problem” shared similar sentence structure and wording. All factor loadings, reported in Table 1, were above 0.50.

Bivariate correlations, reported in Table 2, indicated that coaching relationships were positively correlated with self-efficacy (*r* = 0.60, *p* < 0.001), three dimensions of motivations (perceived benefits *r* = 0.81, *p* < 0.001; low practical reservation *r* = 0.22, *p* < 0.001; low psychological reservation *r* = 0.21, *p* < 0.001), and acceptability of coaching (*r* = 0.90, *p* < 0.001), while negatively correlated with stress (*r* = −0.18, *p* = 0.002). Self-efficacy was positive associated with perceived benefits (*r* = 0.67, *p* < 0.001) and acceptability of coaching (*r* = 0.64, *p* < 0.001), and negatively associated with stress (*r* = −0.20, *p* < 0.001). The three dimensions of motivations (perceived benefits, low practical reservations, low psychological reservations) were all positively correlated with acceptability of coaching (*r*_benefit_ = 0.89, *p* < 0.001; *r*_practical_ = 0.18, *p* < 0.001; *r*_psychological_ = 0.18, *p* = 0.001) and negatively correlated with stress (*r*_benefit_ = −0.19, *p* < 0.001; *r*_practical_ = −0.24, *p* < 0.001; *r*_psychological_ = −0.29, *p* < 0.001). The high correlations between coaching relationship and acceptability of coaching, between perceived benefits and acceptability of coaching suggest some conceptual overlap, thus the heterotrait–monotrait ratio of correlations (HTMT) was computed for all first-order factors to assess the discriminant validity as recommended by Henseler et al. (2015) for variance-based SEM. All HTMT values were below the recommended threshold of 0.90 (Gold et al., 2001), supporting discriminant validity. But it needs to be acknowledged that the HTMT values between coaching relationship and acceptability of coaching (0.89), between perceived benefits and acceptability of coaching (0.87) were approaching but not exceeding the threshold, which was expected given the theoretical connections between these constructs in the school coaching context. These high inter-factor correlations also called for more cautious interpretation of the variances explained in the modeling stage. 

### 3.2. Structural Equation Modeling and Hypothesis Testing

Following the establishment of the measurement model, structural equation modeling (SEM) was conducted to test the theoretically hypothesized model, including specified paths between variables and identified covariates. The model showed acceptable fit: *χ*^2^ (1094) = 1741.59, *p* < 0.001, CFI = 0.92, TLI = 0.91, RMSEA = 0.044, 90% CI [0.040, 0.048], SRMR = 0.063. Using the model trimming approach, non-significant paths were removed after chi-square difference tests indicated no significant change in model fit. The model fit was satisfactory: *χ*^2^ (1099) = 1747.02, *p* < 0.001, CFI = 0.92, TLI = 0.91, RMSEA = 0.044, 90% CI [0.040, 0.048], SRMR = 0.066. In addition, a new path from self-efficacy to stress was added based on a significant chi-square difference test. The final model also showed acceptable fit: *χ*^2^ (1098) = 1738.93, *p* < 0.001, CFI = 0.92, TLI = 0.91, RMSEA = 0.044, 90% CI [0.040, 0.047], SRMR = 0.063. Satorra-Bentler scaled chi-square difference tests were conducted between the full model and the trimmed model (TRd = 5.74, Δ*df* = 5, *p* = 0.33), and between the trimmed model and the final model (TRd = 6.27, Δ*df* = 1, *p* = 0.01). The results showed that the final SEM model fitted significantly better than the trimmed model, while fitting as well as the full model. Furthermore, the final model achieved the lowest AIC and BIC values across three models, indicating its best balance between model fit and parsimony. Our final model explained 38.3% variance in self-efficacy, 70.1% in perceived benefits, 24.0% in practical reservations, 11.6% in psychological reservations, 89.2% in acceptability of coaching, and 22.2% in stress. The high variances explained for perceived benefits and acceptability of coaching might reflect both theoretical connections and potential construct overlap mentioned in the measurement model, which needs to be interpreted with more caution.

**Figure 2:**
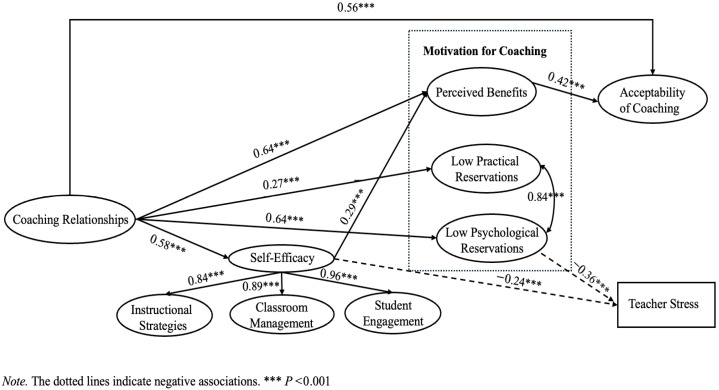
Finalized Model (*N* = 308).

Our final model revealed key relationships between variables of interest (see Table 3 and Figure 2). First, coaching relationships showed positive, significant associations with multiple outcomes, including self-efficacy (β = 0.58, *p* < 0.001, 95%CI [0.49, 0.68]), all three dimensions of motivation: perceived benefits (β = 0.64, , *p* < 0.001, 95%CI [0.51, 0.77]), low practical reservations (β = 0.27, *p* < 0.001, 95%CI [0.18, 0.37]), and low psychological reservations (β = 0.23, *p* < 0.001, 95%CI [0.13, 0.34]), as well as acceptability of coaching (β = 0.56, *p* < 0.001, 95%CI [0.39, 0.72]). The practical and psychological reservations subscales were reverse coded, such that higher scores indicate lower levels of reservation. Therefore, the positive associations between coaching relationships and these dimensions suggest that stronger coaching relationships are linked to reduced practical and psychological reservations about participating in coaching. However, coaching relationships did not have a significant direct association with stress.

Second, self-efficacy was positively associated with perceived benefits (β = 0.29, *p* < 0.001, 95%CI [0.17, 0.41]) as hypothesized. However, it was not significant associated with low practical reservations or low psychological reservations. Although a direct association between self-efficacy and stress was not initially proposed, the model building approach revealed a negative significant path (β = −0.24, *p* < 0.001, 95%CI [−0.36, −0.12]). 

Third, distinct patterns emerged across three dimensions of motivation. Perceived benefits were significantly related to acceptability of coaching (β = 0.42, *p* < 0.001, 95%CI [0.25, 0.59]), while low psychological reservations had a significantly negative association with stress (β = −0.36, *p* < 0.001, 95%CI [−0.48, −0.23]). Low practical reservations, however, were not significantly linked to either acceptability or stress.

### 3.3. Mediation Analysis

The mediation analysis using 5000 bootstrap samples revealed the indirect associations between coaching relationships and both acceptability of coaching and stress, through self-efficacy and motivational factors. In addition to the previously identified direct association, coaching relationships demonstrated a significant total association with the acceptability of coaching (β = 0.89, *p* < 0.001, 95% CI [0.85, 0.94]) and a significant total indirect association (β = 0.34, *p* = 0.001, 95% CI [0.15, 0.53]). The association between coaching relationships and acceptability of coaching was partially explained through perceived benefits (β = 0.27, *p* = 0.003, 95%CI [0.09, 0.45]). Additionally, this association between coaching relationships and acceptability of coaching was sequentially explained through self-efficacy and perceive benefits (β = 0.07, *p* = 0.002, 95%CI [0.03, 0.11]).

Although no direct association was found between coaching relationships and stress, the indirect association was fully explained through self-efficacy (β = −0.14, *p* = 0.002, 95%CI [−0.23, −0.05]) and low psychological reservations (β = −0.08, *p* = 0.003, 95%CI [−0.14, −0.03]). Thus, coaching relationships were negatively associated with stress either through enhanced self-efficacy or low psychological reservation.

## 4. Discussion

This study examined the relationships among coaching relationships, teacher self-efficacy, motivation for coaching, and two key outcomes: teachers’ acceptability of coaching and stress. Additionally, we investigated the roles of self-efficacy and motivation’s three dimensions (i.e., perceived benefits, practical reservations, and psychological reservations) in these associations. To our knowledge, this is one of the few studies to comprehensively explore these interrelated relational and psychological factors within the context of teacher coaching. Theoretically, our findings are consistent with the EVT, particularly in showing that the socializing factor (i.e., coaching relationships) is associated with teachers’ self-efficacy and specific motivation components, which in turn are linked to key outcomes. Practically, the results indicated the importance of fostering strong coaching relationships and addressing motivational barriers to enhance teacher engagement in coaching and reduce stress.

### 4.1. Coaching Relationships as the Socializing Factor

The results of this study consistently highlight the role of coaching relationships and address a recent call to better understand the role of interpersonal dynamics in teacher coaching (Erchul, 2023). First of all, our results indicate that coach–teacher relationship quality is significantly associated with teachers’ acceptability of coaching, consistent with prior research highlighting the importance of relational quality in coaching contexts (Pas et al., 2022; Reddy et al., 2021). While past literature has emphasized the theoretical relevance of coaching relationships in shaping acceptability, this study is among the few to offer empirical evidence supporting that link (Wehby et al., 2012). In addition to its influence on acceptability, the coaching relationship was also found to be strongly and positively associated with teacher self-efficacy. This finding is consistent with the core argument of the EVT, which suggests that when a key socializing figure, such as a coach maintains a supportive and encouraging relationship, individuals are more likely to develop a stronger sense of competence and confidence in their abilities (Eccles & Wigfield, 2023).

Furthermore, our findings demonstrate that coaching relationships are significantly related to all three dimensions of teachers’ motivation for coaching, including perceived benefits, practical reservations, and psychological reservations. Aligning with Johnson et al. (2016), when coaches build strong rapport and trust with teachers, teachers are more likely to see the coaching process as valuable and beneficial. Notably, coaching relationships were also positively associated with lower levels of practical and psychological reservations, suggesting that positive relational dynamics may also help reduce barriers to engagement, such as concerns about time demands or fear of being judged. For example, a teacher who feels respected and supported by their coach may be less anxious about classroom observations and more open to feedback, compared to a teacher who lacks that relational trust. This finding echoes research in the consultation literature that links a consultant’s interpersonal style with teachers’ openness or resistance to change (Blom-Hoffman & Rose, 2007; Newman et al., 2015). Overall, these findings support the hypothesis that coaching relationships are associated with self-efficacy, motivation-related beliefs, and acceptability, suggesting the multifaceted contributions of coaching relationships on teachers’ psychological, attitudinal, and emotional outcomes.

### 4.2. Distinct Roles of Motivational Dimensions

By examining the three distinct dimensions of motivation (i.e., perceived benefits, practical reservations, and psychological reservations), this study offers a more nuanced understanding of how motivation operates in the context of coaching through its distinct components. In light of the growing interest in motivational interviewing within teacher coaching (Lyons et al., 2017; Pas et al., 2022), this study contributes insights into the specific role teachers’ motivation plays in coaching.

First, among the three dimensions, perceived benefits were strongly associated with coaching acceptability, consistent with prior research (Owens et al., 2021). This finding supports EVT’s emphasis on task value as a key correlate of engagement-related outcomes. Moreover, perceived benefits were part of the pattern linking coaching relationships and acceptability, suggesting that teachers who experience a supportive connection with their coach are more likely to see the coaching process as beneficial, which in turn enhances their acceptance of the strategies and feedback offered. 

Second, psychological reservations, which capture discomfort with being observed or fear of negative evaluation, are directly associated with teacher stress. In particular, teachers who experience less emotional hesitation about participating in coaching are more likely to report lower levels of workplace stress, consistent with research suggesting that perceived demands and emotional strain are linked to stress in educational settings (Bottiani et al., 2019). The concept of teachers’ emotional reservations was rarely explored in the teacher coaching literature. This study offers preliminary evidence highlighting how psychological concerns influence teachers’ engagement in coaching and their occupational well-being.

In contrast, practical reservations, which reflect concerns about time, effort, or workload, are not significantly associated with either workplace stress or acceptability of coaching. One possible explanation is that while these concerns are logistically important, they may not exert the same emotional impact as other factors (e.g., fear of judgment or perceived value), and therefore may have a weaker influence on outcomes such as stress or acceptability. As guided by the self-determination theory (Deci & Ryan, 2000), when individuals’ needs for autonomy, competence, and relatedness are fulfilled, they are more intrinsically motivated and less likely to be deterred by external constraints such as time or effort. Thus, the hypothesis regarding the role of all motivational dimensions was only partially supported, suggesting that different components of motivation may play distinct roles.

### 4.3. The Role of Self-Efficacy

Self-efficacy was positively associated with perceived benefits, consistent with prior research linking teacher self-efficacy to autonomous motivation for professional learning through recognition of its inherent value and utility (Zhang et al., 2021). However, no significant associations were found between self-efficacy and practical or psychological reservations. This finding corresponds with Li et al. (2018), who reported that social support-seeking predicted avoidance behaviors, while self-efficacy did not. One possible explanation is that self-efficacy tends to develop through successfully overcoming challenges, which may lead individuals to view situations more positively and thus perceive greater benefits from coaching. However, this positive cognitive appraisal may not necessarily impact an individual’s instinctive tendency to avoid emotionally uncomfortable situations (Carver & Connor-Smith, 2010). While self-efficacy may enhance motivation through cognitive processes, teachers’ practical and psychological reservations (e.g., concerns about time or fear of being judged) are more likely to be influenced by emotional and affective factors than by confidence in their abilities (Li et al., 2018).

Different from the hypothesized model, this study identified a direct association between self-efficacy and stress and found that self-efficacy was linked to the association between coaching relationships and stress. This finding supports prior research indicating that teacher self-efficacy can serve as a buffer against occupational stress (Fu et al., 2022; Herman et al., 2021). Furthermore, corresponding with EVT positing that socializer factors influence motivation through their impact on self-beliefs, the present findings reflect a similar pattern of associations among teachers’ perceptions of coaching relationships, self-efficacy, perceived benefits, and coaching acceptability. Overall, this study provides preliminary correlational evidence suggesting that coaching-related perceptions cluster meaningfully and warrant further investigation using more rigorous longitudinal designs.

### 4.4. Practical Implications

The findings of this study suggest considerations for school coaching initiatives or programs with the goal of increasing teacher acceptance and reducing stress. Given the significant roles of the coaching relationship, we recommend that coaching programs prioritize the cultivation and assessment of high-quality coach-teacher relationships as a foundation for effective coaching. For example, training for coaches could consider including relational competencies such as empathy, active-listening, strength-based feedback, and rapport-building to empower, motivate, and engage teachers. Additionally, measures of coaching fidelity may extend beyond technical implementation to include indicators of coaching relationship quality (Johnson et al., 2016; Pas et al., 2022). Results also indicate the importance of supporting teachers’ self-efficacy within coaching. Coaches may consider actively building teachers’ self-efficacy with a strength-based approach, providing positive and constructive feedback, setting achievable goals, monitoring and praising teacher progress. 

The dimensions of teachers’ motivation for coaching should also be considered in light of the program’s primary goals and objectives. More specifically, the perceived benefits of coaching are linked to acceptability, suggesting that coaches should clearly communicate the purpose and positive impact of coaching, especially for teachers who are not familiar with the process. It might increase teachers’ willingness to participate by framing coaching as a supportive and collaborative process to address challenges they face and enhance teaching efficiency. Results also suggested that teachers with higher psychological reservations (e.g., fear of judgment) reported higher stress. To reduce these concerns, coaches should clarify the confidential nature of the coaching process (Newman & Rosenfield, 2024), assuring teachers that their performance will not be shared with administrators. Since different motivational components (e.g., perceived benefits, practical reservations, and psychological reservations) have varying relationships with outcomes, it may be helpful for school leaders and coaches to consider assessing teachers’ motivational profiles (Owens et al., 2021; Wang et al., 2025a).

### 4.5. Limitations and Future Study Directions

One limitation of this study is its cross-sectional design, which prevents causal interpretations among variables. Taris et al. (2021) recommended collecting data on both antecedent and outcome variables at more than one time point to help draw causal inferences. Future research could adopt a longitudinal approach, such as a time-series study, by tracking teachers before, during, and after coaching interventions to establish causality and assess the impact of various coaching factors. The gender distribution of the teacher sample in this study is skewed toward male and was not stratified across regions of the United States, which may reflect potential sampling bias due to online panel recruitment and limit the generalizability of the findings.

Additionally, this study relied on self-report measures collected from teachers. For example, teachers’ perceptions of coaching acceptability reflect their general attitudes toward coaching based on cumulative experiences rather than specific coaching interaction with defined duration or frequency. Similarly, assessments of coaching relationships were based on teachers’ retrospective views of their interactions with coaches. While these self-report measures provide valuable insights into teachers’ perspectives and can offer broad generalizability, they lack objective indicators for specific coaching experiences. Future research should consider incorporating objective measures or observational methods within the context of specific coaching programs. For instance, researchers could collect the frequency with which teachers actively seek coaching support as indicators of their acceptability of coaching. 

Given the significant role that coaching relationship plays in the success of coaching interventions, more research is needed to better understand how to effectively build and sustain positive coaching relationships with teachers. This study also calls for further investigation into the roles of self-efficacy and motivation in coaching contexts. In addition to acceptability and stress, future studies should examine the impact of these psychological factors on outcomes such as teachers’ intervention fidelity, teacher retention, and student outcomes, providing a more comprehensive understanding of how coaching supports both teacher development and broader educational goals.

Furthermore, the high correlations among several latent constructs, specifically coaching relationships, perceived benefits, and acceptability of coaching acceptability, propose concerns about conceptual overlapping. Therefore, the interpretation of structural paths and variances explained in the outcomes, particularly acceptability of coaching, should be approached with caution. Future studies could utilize replication study to further validate the discriminant validity of these constructs. Next, although the single-item measure of stress has been validated by Eddy et al. (2019), it is more vulnerable to measurement error and might not capture the construct adequately (Allen et al., 2022). Thus, the conclusions related to teacher stress should be interpreted more cautiously and future studies should employ multi-item measures of stress to provide more precise estimates. Finally, the model included partial reliance on post hoc modifications based on modification fit indices, given the limited literature on these associations in the context of coaching. Although these modifications were informed by conceptual considerations, they may limit the generalizability of the model and should be validated in future research.

## 5. Conclusions

This study examined the associations among coaching relationships, teacher self-efficacy, motivation for coaching, teachers’ acceptability of coaching, and perceived stress. The findings provide preliminary evidence that these relational and psychological factors are meaningfully associated, with coaching relationships linked to self-efficacy and motivation-related beliefs, which in turn are associated with acceptability and stress. Consistent with EVT, the results suggest that socializing factor (i.e., coaching relationship) is related to teachers’ self-efficacy, motivation, and performance outcomes.

## Figures and Tables

**Table 1:** Descriptive Statistics of Latent Variables (*N* = 308).

Variable		Item	Factor Loading	*M*	*SD*	α	CR	AVE
Coaching Relationships		The coach and I agreed on what the most important goals for intervention were.	0.81	4.24	0.70	0.87	0.88	0.56
		The coach and I trusted one another.	0.71					
		The coach was approachable.	0.71					
		The coach and I worked together collaboratively.	0.78					
		Overall, the coach showed a sincere desire to understand and improve my classroom.	0.79					
		The coach incorporated my views into the services provided.	0.69					
Self-Efficacy	Instructional Strategies	To what extent can you use a variety of assessment strategies?	0.62	7.50	1.34	0.70	0.71	0.45
		To what extent can you craft good questions for your students?	0.72					
		How well can you implement alternative strategies in your classroom?	0.68					
	Classroom Management	How much can you do to control disruptive behavior in the classroom?	0.76	7.42	1.32	0.75	0.76	0.51
		How much can you do to get children to follow classroom rules?	0.72					
		How much can you do to calm a student who is disruptive or noisy?	0.67					
	Student Engagement	How much can you do to get students to believe they can do well in schoolwork?	0.69	7.37	1.36	0.81	0.81	0.53
		How much can you do to help your students value learning?	0.72					
		How much can you do to motivate students who show low interest in schoolwork?	0.67					
		How much can you assist families in helping their children do well in school?	0.62					
Perceived Benefits		I appreciate the coach’s help in addressing my concerns about students	0.61	4.27	0.67	0.92	0.92	0.53
		The coach shares helpful materials and resources for teaching with me	0.63					
		Coaching helps me assist students with lower academic performance	0.73					
		Strategies I learn during coaching sessions help me become a better teacher	0.73					
		The coach helps me to identify my students’ needs	0.78					
		Coaching helps me manage student behaviors	0.77					
		The coach helps me review student progress throughout the coaching sessions	0.68					
		Coaching helps me improve my interactions with students	0.79					
		The coach helps me set up clear goals for our coaching sessions	0.77					
		I am motivated to achieve the goals that I identified with my coach	0.68					
Practical Reservations		* Coaching does not make a difference to me in terms of my teaching practice	0.68	3.34	1.12	0.83	0.84	0.57
		* The goals that my coach and I set are unrealistic	0.68					
		* Explaining student concerns to my coach takes too much effort	0.77					
		* The effort that it takes to participate in coaching is not worth the benefits	0.86					
Psychological Reservations		* I am worried about being evaluated negatively by a coach	0.78	3.00	1.01	0.88	0.89	0.59
		* I feel stressed when the coach asks me to use teaching strategies that I am not familiar with	0.78					
		* I feel nervous about being observed by the coach	0.77					
		* I am worried the coach will judge me	0.83					
		* It is stressful to follow the action plans set up for coaching	0.75					
		* I am too tired during the school day to receive coaching	0.66					
Acceptability of Coaching		The coaching is an effective choice for addressing a variety of problems.	0.82	4.87	0.99	0.92	0.93	0.59
		The coaching is a fair way to handle the child’s behavior problem.	0.78					
		* I would not be interested in receiving coaching.	0.85					
		I would have positive attitudes about receiving coaching.	0.82					
		Coaching is a good way to handle the child’s behavior problem	0.72					
		I would receive coaching with a good deal of enthusiasm.	0.83					
		Coaching would not be disruptive to other students.	0.52					
		I would be committed to receiving coaching.	0.79					
		The coaching procedures easily fit in with my current practices.	0.86					
Stress		How stressful is your job?	1	6.65	2.60	na	na	na

*Note.* * Denotes reverse-scored items. na = not applicable.

**Table 2:** Bivariate Correlations Between Latent Variables (*N* = 308).

	1	2	3	4	5	6	7
1. Coaching Relationships							
2. Self-Efficacy	0.61 ***						
3. Perceived Benefits	0.81 ***	0.67 ***					
4.Low Practical Reservations	0.22 ***	0.04	0.22 ***				
5. Low psychological Reservations	0.21 ***	0.04	0.18 ***	0.85 ***			
6. Acceptability of Coaching	0.90 ***	0.64 ***	0.88 ***	0.19 ***	0.18 ***		
7. Stress	−0.18 ***	−0.19 ***	−0.19 ***	−0.24 ***	−0.29 ***	−0.15 **	

*Note.* ** *p* < 0.01. *** *p* < 0.001. Practical Reservations and Psychological Reservations were reversed coded with higher scores indicating lower reservations. na = not applicable.

**Table 3:** Direct and Indirect Results for the Final Model (*N* = 308).

Direct Associations	*B*	β	*SE* (*β*)	*p*	95%CI (*β*)
Self-Efficacy ON Coaching Relationships	0.73	0.58	0.05	<0.001	[0.49, 0.68]
Perceived Benefits ON					
Coaching Relationships	0.41	0.64	0.07	<0.001	[0.51, 0.77]
Self-Efficacy	0.15	0.29	0.06	<0.001	[0.17, 0.41]
Low Practical Reservations ON Coaching Relationships	0.38	0.27	0.05	<0.001	[0.18, 0.37]
Low Psychological Reservations ON Coaching Relationships	0.35	0.23	0.05	<0.001	[0.13, 0.34]
Acceptability of Coaching ON					
Coaching Relationships	0.74	0.56	0.08	<0.001	[0.39, 0.72]
Perceived Benefits	0.88	0.42	0.09	<0.001	[0.25, 0.59]
Stress ON					
Self-Efficacy	−0.54	−0.24	0.06	<0.001	[−0.36, −0.12]
Low Psychological Reservations	−0.67	−0.36	0.06	<0.001	[−0.48, −0.23]
**Indirect Associations**					
Coaching Relationships To Acceptability of Coaching					
Total Effect	1.19	0.89	0.02	<0.001	[0.85, 0.94]
Total Indirect	0.45	0.34	0.10	0.001	[0.15, 0.53]
Path 1: Coaching Relationships → Perceived Benefit → Acceptability	0.36	0.27	0.09	0.003	[0.09, 0.45]
Path 2: Coaching Relationships → Self-Efficacy → Perceived Benefit → Acceptability	0.09	0.07	0.02	0.002	[0.03, 0.11]
Direct	0.74	0.56	0.08	<0.001	[0.35, 0.76]
Coaching Relationships to Stress					
Total Effect	−0.63	−0.23	0.05	<0.001	[−0.32, −0.12]
Total Indirect	−0.63	−0.23	0.05	<0.001	[−0.32, −0.12]
Path 1: Coaching Relationship → Low Psychological Reservations → Stress	−0.23	−0.08	0.03	0.003	[−0.14, −0.03]
Path 2: Coaching Relationships → Self-Efficacy → Stress	−0.39	−0.14	0.04	0.002	[−0.23, −0.05]
**Significant Covariates**					
Coaching Relationships ON Gender	0.22	0.15	0.05	0.003	[0.05, 0.25]
Self-Efficacy ON Gender	0.25	0.14	0.04	0.001	[0.06, 0.22]
Low Practical Reservations ON					
Gender	−0.74	−0.38	0.05	<0.001	[−0.47, −0.29]
Age	0.04	0.30	0.05	<0.001	[0.19, 0.40]
Years of Teaching	−0.03	−0.25	0.06	<0.001	[−0.36, −0.15]
Low Psychological Reservations ON					
Gender	−0.44	−0.21	0.05	<0.001	[−0.30, −0.12]
Age	0.03	0.23	0.06	<0.001	[0.10, 0.35]
Years of Teaching	−0.03	−0.21	0.06	0.001	[−0.33, −0.08]
Acceptability of Coaching ON Gender	0.15	0.08	0.03	0.001	[0.03, 0.13]
Stress ON Years of Teaching	0.04	0.09	0.04	0.02	[0.01, 0.17]

## Data Availability

The data presented in this study are available on request from the corresponding author.

## References

[B1-behavsci-16-00838] Alavi M., Visentin D. C., Thapa D. K., Hunt G. E., Watson R., Cleary M. (2020). Chi-square for model fit in confirmatory factor analysis. Journal of Advanced Nursing.

[B2-behavsci-16-00838] Allen M. S., Iliescu D., Greiff S. (2022). Single item measures in psychological science. European Journal of Psychological Assessment.

[B3-behavsci-16-00838] Aytaç A. (2021). A Study of teachers’ self-efficacy beliefs, motivation to teach, and curriculum fidelity: A path analysis model. International Journal of Contemporary Educational Research.

[B4-behavsci-16-00838] Bandura A. (1999). Social cognitive theory: An agentic perspective. Asian Journal of Social Psychology.

[B5-behavsci-16-00838] Bandura A. (2006). Toward a psychology of human agency. Perspectives on Psychological Science.

[B6-behavsci-16-00838] Blom-Hoffman J., Rose G. S. (2007). Applying motivational interviewing to school-based consultation: A commentary on “has consultation achieved its primary prevention potential”?. Journal of Educational and Psychological Consultation.

[B7-behavsci-16-00838] Bottiani J. H., Duran C. A., Pas E. T., Bradshaw C. P. (2019). Teacher stress and burnout in urban middle schools: Associations with job demands, resources, and effective classroom practices. Journal of School Psychology.

[B8-behavsci-16-00838] Briesch A. M., Chafouleas S. M., Neugebauer S. R., Riley-Tillman T. C. (2013). Assessing influences on intervention implementation: Revision of the Usage Rating Profile-Intervention. Journal of School Psychology.

[B9-behavsci-16-00838] Calkins L., Wiens P., Parker J., Tschinkel R. (2024). Teacher motivation and self-efficacy: How do specific motivations for entering teaching relate to teacher self-efficacy?. Journal of Education.

[B10-behavsci-16-00838] Carver C. S., Connor-Smith J. (2010). Personality and coping. Annual Review of Psychology.

[B11-behavsci-16-00838] Cohen S., Wills T. A. (1985). Stress, social support, and the buffering hypothesis. Psychological Bulletin.

[B12-behavsci-16-00838] Çoban Ö., Özdemir N., Bellibaş M. Ş. (2020). Trust in principals, leaders’ focus on instruction, teacher collaboration, and teacher self-efficacy: Testing a multilevel mediation model. Educational Management Administration & Leadership.

[B13-behavsci-16-00838] Dart E. H., Cook C. R., Collins T. A., Gresham F. M., Chenier J. S. (2012). Test driving interventions to increase treatment integrity and student outcomes. School Psychology Review.

[B14-behavsci-16-00838] Davis A. E., Barrueco S., Perry D. F. (2021). The role of consultative alliance in infant and early childhood mental health consultation: Child, teacher, and classroom outcomes. Infant Mental Health Journal.

[B15-behavsci-16-00838] Deci E. L., Ryan R. M. (2000). The “what” and “why” of goal pursuits: Human needs and the self-determination of behavior. Psychological Inquiry.

[B16-behavsci-16-00838] Doménech-Betoret F., Abellán-Roselló L., Gómez-Artiga A. (2017). Self-efficacy, satisfaction, and academic achievement: The mediator role of students’ expectancy-value beliefs. Frontiers in Psychology.

[B17-behavsci-16-00838] Eccles J. S., Wigfield A. (2023). Expectancy-value theory to situated expectancy-value theory: Reflections on the legacy of 40+ years of working together. Motivation Science.

[B18-behavsci-16-00838] Eddy C. L., Herman K. C., Reinke W. M. (2019). Single-item teacher stress and coping measures: Concurrent and predictive validity and sensitivity to change. Journal of School Psychology.

[B19-behavsci-16-00838] Erchul W. P. (2023). As we coach, so shall we consult: A perspective on coaching research in education. Journal of School Psychology.

[B20-behavsci-16-00838] Frank J. L., Kratochwill T. R., Erchul W. P., Sheridan S. M. (2014). School-based problem-solving consultation: Plotting a new course for evidence-based research and practice in consultation. Handbook of research in school consultation.

[B21-behavsci-16-00838] Fu W., Wang L., He X., Chen H., He J. (2022). Subjective well-being of special education teachers in China: The relation of social support and self-efficacy. Frontiers in Psychology.

[B22-behavsci-16-00838] Gold A. H., Malhotra A., Segars A. H. (2001). Knowledge management: An organizational capabilities perspective. Journal of Management Information Systems.

[B23-behavsci-16-00838] Haydon T., Leko M. M., Stevens D. (2018). Teacher stress: Sources, effects, and protective factors. Journal of Special Education Leadership.

[B24-behavsci-16-00838] Henseler J., Ringle C. M., Sarstedt M. (2015). A new criterion for assessing discriminant validity in variance-based structural equation modeling. Journal of the Academy of Marketing Science.

[B25-behavsci-16-00838] Herman K. C., Sebastian J., Reinke W. M., Huang F. L. (2021). Individual and school predictors of teacher stress, coping, and wellness during the COVID-19 pandemic. School Psychology.

[B26-behavsci-16-00838] Hu L., Bentler P. M. (1999). Cutoff criteria for fit indexes in covariance structure analysis: Conventional criteria versus new alternatives. Structural Equation Modeling.

[B27-behavsci-16-00838] Jentsch A., Hoferichter F., Blömeke S., König J., Kaiser G. (2023). Investigating teachers’ job satisfaction, stress and working environment: The roles of self-efficacy and school leadership. Psychology in the Schools.

[B28-behavsci-16-00838] Johnson S., Pas E., Bradshaw C., Johnson S. R., Pas E. T., Bradshaw C. P. (2016). Understanding and measuring coach-teacher alliance: A glimpse inside the “black box”. Prevention Science.

[B29-behavsci-16-00838] Kazdin A. E. (1980). Acceptability of alternative treatments for deviant child behavior. Journal of Applied Behavior Analysis.

[B30-behavsci-16-00838] Kenny D. A. (2012). Identification.

[B31-behavsci-16-00838] Kingsford-Smith A. A., Collie R. J., Loughland T., Nguyen H. T. M. (2023). Teacher wellbeing in rural, regional, and metropolitan schools: Examining resources and demands across locations. Teaching and Teacher Education.

[B32-behavsci-16-00838] Kline R. B. (2016). Principles and practice of structural equation modeling.

[B33-behavsci-16-00838] Kochmanski N. (2023). Identifying productive one-on-one coaching practices. Teacher and Teacher Education.

[B34-behavsci-16-00838] Kraft M. A., Blazar D., Hogan D. (2018). The effect of teacher coaching on instruction and achievement: A meta-analysis of the causal evidence. Review of Educational Research.

[B35-behavsci-16-00838] Li M. H., Eschenauer R., Persaud V. (2018). Between avoidance and problem solving: Resilience, self-efficacy, and social support seeking. Journal of Counseling & Development.

[B36-behavsci-16-00838] Lyons M. D., Jones S. J., Smith B. H., McQuillin S. D., Richardson G., Reid E., McClellan A. (2017). Motivation coaching training for instructional coaches: A pilot study of motivational interviewing skills training. Mentoring & Tutoring: Partnership in Learning.

[B37-behavsci-16-00838] Marsh H. W., Hau K.-T., Grayson D., Maydeu-Olivares A., McArdle J. (2005). Goodness of fit evaluation. Contemporary psychometrics.

[B38-behavsci-16-00838] Maslach C., Jackson S. E., Schwab R. L., Maslach C., Jackson S. E., Leiter M. P. (1996). Maslach burnout inventory—Educators survey (MBI-ES). MBI manual.

[B39-behavsci-16-00838] Merle J. L., Cook C. R., Pullmann M. D., Larson M. F., Hamlin C. M., Hugh M. L., Brewer S. K., Duong M. T., Bose M., Lyon A. R. (2023). Longitudinal effects of a motivationally focused strategy to increase the yield of training and consultation on teachers’ adoption and fidelity of a universal program. School Mental Health.

[B40-behavsci-16-00838] Moshagen M., Bader M. (2024). semPower: General power analysis for structural equation models. Behavior Research Methods.

[B41-behavsci-16-00838] Neff K. D., Tóth-Király I., Yarnell L. M., Arimitsu K., Castilho P., Ghorbani N., Guo H. X., Hirsch J. K., Hupfeld J., Hutz C. S., Kotsou I., Lee W. K., Montero-Marin J., Sirois F. M., de Souza L. K., Svendsen J. L., Wilkinson R. B., Mantzios M. (2019). Examining the factor structure of the self-compassion scale in 20 diverse samples: Support for use of a total score and six subscale scores. Psychological Assessment.

[B42-behavsci-16-00838] Newman D. S., Guiney M. C., Barrett C. A. (2015). Language use in consultation: Can “we” help teachers and students?. Consulting Psychology Journal.

[B43-behavsci-16-00838] Newman D. S., Rosenfield S. A. (2024). Building competence in school consultation: A developmental approach.

[B44-behavsci-16-00838] Osman D. J., Warner J. R. (2020). Measuring teacher motivation: The missing link between professional development and practice. Teaching and Teacher Education.

[B45-behavsci-16-00838] Owens J. S., Lee M., Kassab H., Evans S. W., Coles E. C. (2021). Motivational ruler ratings among teachers receiving coaching in classroom management: Measurement and relationship to implementation integrity. Prevention Science.

[B46-behavsci-16-00838] Palermo C., Thomson M. M. (2019). Large-scale assessment as professional development: Teachers’ motivations, ability beliefs, and values. Teacher Development.

[B47-behavsci-16-00838] Pas E. T., Borden L., Debnam K. J., De Lucia D., Bradshaw C. P. (2022). Exploring profiles of coaches’ fidelity to double check’s motivational interviewing-embedded coaching: Outcomes associated with fidelity. Journal of School Psychology.

[B48-behavsci-16-00838] Pollfish (2025). Online sampling service service.

[B49-behavsci-16-00838] Reddy L. A., Glover T. A., Dudek C. M., Alperin A., Wiggs N. B., Bronstein B. (2022). A randomized trial examining the effects of paraprofessional behavior support coaching for elementary students with disruptive behavior disorders: Paraprofessional and student outcomes. Journal of School Psychology.

[B50-behavsci-16-00838] Reddy L. A., Glover T. A., Elliott S. N., Kurz A. (2019). Assessing the effectiveness and interactions of instructional coaches: Initial psychometric evidence for the instructional coaching assessments–teacher form. Assessment for Effective Intervention.

[B51-behavsci-16-00838] Reddy L. A., Shernoff E., Lekwa A. (2021). A randomized controlled trial of instructional coaching in high-poverty urban schools: Examining teacher practices and student outcomes. Journal of School Psychology.

[B52-behavsci-16-00838] Ruble L., Ogle L., McGrew J. (2023). Practice makes proficient: Evaluation of implementation fidelity following COMPASS consultation training. Psychology in the Schools.

[B53-behavsci-16-00838] Ryan R. M., Deci E. L. (2000). Self-determination theory and the facilitation of intrinsic motivation, social development, and well-being. American Psychologist.

[B54-behavsci-16-00838] Shernoff E. S., Lekwa A. J., Reddy L. A., Coccaro C. (2017). Examining teachers’ attitudes and experiences with coaching to inform research-based practice: An iterative developmental design study. Journal of Educational & Psychological Consultation.

[B55-behavsci-16-00838] Skaalvik E. M., Skaalvik S. (2017). Motivated for teaching? Associations with school goal structure, teacher self-efficacy, job satisfaction and emotional exhaustion. Teaching and Teacher Education.

[B56-behavsci-16-00838] Steigleder J., Buhr L., Ehm J.-H., Gawrilow C., von Suchodoletz A. (2023). Changes in subjective stress experiences and self-efficacy beliefs of preschool teachers in Germany: A longitudinal study during 12 months of the COVID-19 pandemic. Teaching and Teacher Education.

[B57-behavsci-16-00838] Sun Y., Yin H. (2025). Profiles of teacher self-efficacy and their relations to teacher demographics and affective well-being: A social cognitive perspective. Teaching and Teacher Education.

[B58-behavsci-16-00838] Taris T. W., Kessler S. R., Kelloway E. K. (2021). Strategies addressing the limitations of cross-sectional designs in occupational health psychology: What they are good for (and what not). Work & Stress.

[B59-behavsci-16-00838] Teng M. F., Wang C., Wu J. G. (2021). Metacognitive strategies, language learning motivation, self-efficacy belief, and English achievement during remote learning: A structural equation modelling approach. RELC Journal.

[B60-behavsci-16-00838] Toto G. A., Limone P. (2021). Motivation, stress and impact of online teaching on Italian teachers during COVID-19. Computers.

[B61-behavsci-16-00838] Tschannen-Moran M., Hoy A. W. (2001). Teacher efficacy: Capturing an elusive construct. Teaching and Teacher Education.

[B62-behavsci-16-00838] Urhahne D., Wijnia L. (2023). Theories of motivation in education: An integrative framework. Educational Psychology Review.

[B63-behavsci-16-00838] Wang J., Kalkbrenner M. T., Begeny J. C. (2025a). Development and initial validation of scores on the teacher’s motivation assessment for coaching (T-MAC). Journal of Educational and Psychological Consultation.

[B64-behavsci-16-00838] Wang J., Lee B. H., Zhao Y. (2025b). Examining relationships of country, school context, and coping on teacher stress in the United States and the United Kingdom. International Journal of School & Educational Psychology.

[B65-behavsci-16-00838] Wehby J. H., Maggin D. M., Moore Partin T. C., Robertson R. (2012). The impact of working alliance, social validity, and teacher burnout on implementation fidelity of the good behavior game. School Mental Health.

[B66-behavsci-16-00838] Wigfield A., Eccles J. S. (2000). Expectancy–value theory of achievement motivation. Contemporary Educational Psychology.

[B67-behavsci-16-00838] Yin H., Guo Y., Liu Z. (2025). Teachers as thinkers, not feelers: Clarifying the definition and measurement of attitude in teachers’ acceptance of online teaching. Teaching and Teacher Education.

[B68-behavsci-16-00838] Zhang X., Admiraal W., Saab N. (2021). Teachers’ motivation to participate in continuous professional development: Relationship with factors at the personal and school level. Journal of Education for Teaching.

